# Healing with Risks: How Zoonotic Potential Influences the Use of Wild Mammals in Traditional Medicine

**DOI:** 10.3390/pathogens14070640

**Published:** 2025-06-27

**Authors:** Heliene Mota Pereira, Mayara Guimarães Beltrão, Anna Karolina Martins Borges, Weslley Ruan Guimarães da Silva, Danilo Vicente Batista Oliveira, Rômulo Romeu Nóbrega Alves

**Affiliations:** 1 Programa de Pós-Graduação em Etnobiologia e Conservação da Natureza, Universidade Federal Rural de Pernambuco, Recife 52171-900, Brazil; karolm26@hotmail.com; 2 Laboratório de Biologia da Conservação, Departamento de Biodiversidade, Universidade Estadual Paulista “Júlio de Mesquita Filho”, Rio Claro 13506-900, Brazil; mayarabeltrao@gmail.com; 3 LAPEC—Laboratório de Peixes e Conservação Marinha, Universidade Estadual da Paraíba, João Pessoa 58071-160, Brazil; 4 Bioinformatics Multidisciplinary Environment, BioME, Federal University of Rio Grande do Norte, Natal 59000-000, Brazil; weslley.silva.128@ufrn.edu.br; 5 ESG e Compliance, Masterboi, Av. da Recuperação, 7380 Dois Irmãos, Recife 52171-340, Brazil; dnlvbo@gmail.com; 6 Centro de Ciências Biológicas e Sociais Aplicadas, Universidade Estadual da Paraíba, João Pessoa 58071-160, Brazil; rrnalves@servidor.uepb.edu.br

**Keywords:** medicinal animals, human–animal interactions, zoonoses, ethnozoology, zootherapy

## Abstract

Most infectious diseases affecting humans are zoonotic in origin, with mammals serving as the main reservoirs. Frequent interactions between humans and animals, especially in the context of their use for food, medicine, and other purposes, pose significant public health risks, as recently demonstrated by the SARS-CoV-2 pandemic. In traditional medicine, many species—some of which are also used as food—are valued for their therapeutic versatility, that is, the diversity of medicinal uses attributed to each species. This study investigates the role of zoonotic potential in the selection of mammals used in traditional medicine at a global scale. We compiled data on 411 wild mammal species across 17 orders, identifying 5.146 associated pathogens, of which 2.778 (53.9%) also infect humans. Most diseases transmitted by these species are caused by viruses (33.4%), bacteria (23.3%), and helminths (22.3%). These mammals are used to treat at least 500 diseases or symptoms, and 4.3% of the species show high therapeutic versatility (RI > 1). Our results indicate that species selection is shaped by both biological and cultural factors, with zoonotic potential being the most influential: species with a higher risk of disease transmission tend to be less used. These findings highlight the importance of incorporating zoonotic risk into research and policies regarding the medicinal use of wildlife.

## 1. Introduction

Emerging infectious diseases, especially zoonotic ones, are a global concern due to their impact on public health. The COVID-19 pandemic, caused by the SARS-CoV-2 virus, illustrates how such diseases can lead to significant global health, economic and social disruptions. Zoonotic pathogens account for approximately 60% of known human infections and 75% of emerging infectious diseases, and are often associated with the global wildlife trade [1,2,3]. Part of this trade involves the use of wild animals in traditional medicine, a practice shaped by cultural and economic factors, which often contributes to illegal hunting [4,5,6].

Human contact with wild animals during hunting, slaughter, transport and consumption increases the risk of zoonosis transmission [4,7,8,9]. Bites, scratches and exposure to bodily fluids can facilitate infection, especially when injuries are caused during handling [10]. Medicinal uses include organs, fat, blood, skins, feathers, bones, glands and excrement, which can serve as potential sources of disease [10,11,12,13]. Recurrent exposure to wild animals and their bodily fluids can drive evolutionary adaptations in pathogens, increasing the likelihood of zoonotic spillover, defined as the transmission of animal-derived pathogens to humans [14,15]. Despite the risks involved, the use of wild fauna represents an important therapeutic alternative in many traditional medical systems around the world, especially in contexts of low availability or limited access to conventional drugs [16].

Mammals are often associated with the emergence of zoonoses due to evolutionary, biological and ecological factors which favor the transmission of pathogens [1,9,17,18,19]. Diseases such as Ebola, Influenza, Mpox, Plague, Acquired Immunodeficiency Syndrome (AIDS) and COVID-19 illustrate the recurrence of zoonotic emergencies in recent decades, reinforcing the need for epidemiological investigations [20]. In this scenario, monitoring human exposure to zoonoses associated with traditional medicine and other cultural practices is a relevant strategy for anticipating risks to public health [21,22]

Selecting animals for medicinal use involves myths, popular beliefs and traditional knowledge, along with characteristics of the species such as body mass, feeding habits, behavior and their accessibility [23,24,25]. These factors are dynamic and can be modified as new species are incorporated into human consumption [26]. The medicinal importance of a species can be assessed by its versatility, meaning by the number of therapeutic indications associated with it [24,27,28], which may also have implications for conservation and bioprospecting [29].

Although it remains unclear how the zoonotic potential influences species selection in traditional medicine, from an adaptive perspective, one might expect individuals to avoid species perceived as risky, even without formal scientific knowledge. Behavioral mechanisms such as disgust or aversion to certain animal traits, often shaped by empirical observation and cultural transmission, may act as proxies for risk, contributing to zoonosis avoidance [30,31]. However, traditional medical systems are shaped by a complex web of ecological, cultural and symbolic factors [32,33].

Biological and ecological aspects can be reflected in cultural perceptions, influencing the acceptance or rejection of species as therapeutic resources in zootherapeutic practices across different sociocultural contexts [34]. Zoonotic potential and ecological attributes, such as feeding habits and body mass, are recognized as relevant factors in the selection and valuation of species in traditional contexts, and may, either individually or in combination, help explain their therapeutic versatility. Large animals are more likely to host and transmit pathogens due to their greater longevity, large home range and greater complexity in ecological interactions [35,36]. Furthermore, dietary habits influence this risk: carnivorous and omnivorous species are more likely to be exposed to and infected by pathogens when consuming infected prey, while herbivores tend to have a reduced zoonotic risk [37].

This study uses medicinal mammals as a model to investigate how ecological characteristics relate to zoonotic risk and therapeutic versatility. Specifically, we seek to (1) assess the spatial distribution of zoonotic potential among wild medicinal mammals, and (2) examine how therapeutic versatility (defined as the range of medicinal uses attributed to an animal) is influenced by zoonotic potential, body mass, and feeding habits.

Although traditional knowledge systems do not use formal epidemiological indicators, perceptions of disease risk may arise from empirical observations, symbolic associations, and cultural transmission. Animals seen as unclean, aggressive, or foul-smelling are often avoided, reflecting intuitive strategies of risk avoidance. This aligns with the behavioral immune system theory, which highlights evolved responses like disgust to prevent infection [38] and has been observed in both primate behavior and traditional human contexts [39]. Based on this evidence, we hypothesize that species with higher zoonotic potential are less frequently used in traditional medicine, due to empirical perceptions of health risk, regardless of their therapeutic versatility, while species with greater body mass or herbivorous diets may be more versatile due to both practical (such as biomass availability) and cultural criteria (such as symbolisms associated with the diet or size of the animals) [6,24,32,33,34,35,36,37,38,39].

## 2. Materials and Methods

### 2.1. Data Collection

The data for this study were compiled from several previous investigations. First, we retrieved records from the database which records medicinal species and their respective uses [24]. We searched for host–parasite relationship data for all mammals using the “Mammal-pathogen-species-level associations” dataset [40] and identified which pathogens were zoonotic through the “SpeciesInteractions_EID2” resource [41]. Zoonotic status was determined computationally in R by matching pathogens associated with humans (*Carrier.classification* = “Human”) to the mammal–pathogen records. Both datasets were accessed programmatically in R (version 4.4.1) in April 2022.

We checked whether species were classified as domestic according to the list provided by the Food and Agriculture Organization (FAO) [42]. To standardize and harmonize the scientific names in the final dataset, we employed the R package Taxize version 4.4.1 [43], using the ‘classification’ function to retrieve hierarchical taxonomic records via the Global Biodiversity Information Facility (GBIF) [44], ensuring alignment with current and widely accepted classifications. This process allowed us to correct outdated names, resolve synonyms, and consistently assign each species to its respective genus, family and order, thus improving taxonomic accuracy and ecological comparability across analyses.

We subsequently compiled information on body mass (g) from the PanTHERIA database [45]. Data on dietary habits were collected from various published sources, including PanTHERIA [45], ANIMALIA [46] and GBIF [44]. We classified host mammals into four categories of feeding habits: herbivores, carnivores, insectivores, and omnivores. Information on the geographic distribution of species was obtained from the International Union for Conservation of Nature (IUCN) [47] database. To do this, we overlapped the IUCN species occurrence maps with the list of mammals used in traditional medicine compiled by [24], using scientific names as a matching criterion. For the spatial analyses, when the overlap of host mammal species distribution maps resulted in multiple distinct areas, only the largest area of occurrence was retained for each species. The usage frequency of each species by country was measured as the total number of independent medicinal use records documented in each country, based on our dataset (Table S1).

### 2.2. Data Analysis

First, we quantified the total pathogen richness by compiling the number of unique pathogen species reported for each mammalian species used in traditional medicine. We then estimated the zoonotic potential based on the number of pathogens shared between each mammalian species and humans, thus characterizing the zoonotic richness [36]. Finally, both values were aggregated by taxonomic order, resulting in estimates of total and zoonotic pathogen richness for each group.

We used the therapeutic versatility of the species as a response variable in our models. In this study, we define therapeutic versatility as the diversity of medicinal uses attributed to a species. This concept is operationalized through the Relative Importance Index (RI), which is used to identify the most versatile species considering the number of medicinal properties (uses) reported by informants [48]. The RI was calculated using the formula RI = NUC + NT, in which NUC represents the ratio between the number of disease categories associated with a species (NUCS) and the total number of disease categories of the most versatile species (NUCVS), while NT represents the number of types of uses attributed to each species (NTS). The RI value ranges from 0 (no medicinal use) to 2 (maximum versatility). Although cultural and symbolic values influence species selection, our statistical modeling focused on ecological and zoonotic variables due to the lack of standardized data across species and regions. These aspects are addressed qualitatively in the discussion.

Before fitting the models, we pre-treated the variables to ensure compliance with the statistical assumptions and improve interpretation of the results. The numerical variables (body mass and zoonotic potential) were log_10_-transformed to reduce skewness and stabilize variance. In addition, we transformed the continuous variable *body mass*, originally expressed in grams (g). Subsequently, the variables were rescaled (standardized) to meet the assumptions of the statistical models and to facilitate the interpretation of parameter estimates.

To control for potential inflation of versatility values in well-studied species, we adjusted the Relative Importance Index (RI) by dividing it by the number of bibliographic records per species. This correction accounts for uneven research effort and improves the comparability of versatility estimates across species. The resulting value represents the corrected versatility, referred to from now on as “versatility weighted”. This variable was adjusted to minimize the impact of possible inequalities. The results of these values are presented in Table S1.

Next, we tested the data normality and checked the variance inflation factor (VIF) to detect whether there was collinearity between the predictor variables. Based on the values (all below the threshold of 3), we verified that there were no multicollinearity problems in the model [49]. We used the residuals of a multiple linear regression model evaluating the effects of the predictor variables on versatility weighted to verify whether there was the presence of spatial autocorrelation using the testSpatialAutocorrelation function of the DHARMa package (version 0.4.6) [50].

Due to detecting spatial autocorrelation in the data, we used a mixed additive model fitted by approximate maximum likelihood to evaluate the relationship between therapeutic versatility (=versatility weighted) and its potential explanatory factors, implemented through the fitme function of the spaMM package in R [51]. The response variable (=versatility weighted) was modeled using a Gamma distribution and a logarithmic link Γ(link = log), appropriate for positive and asymmetric data. In the general model, the predictor variables included zoonotic potential, body mass and feeding habits as fixed covariates with additive relations. Based on this, the remaining models were constructed, including three univariate models, three bivariate models with additive relationships, and one null model. Then, we included a spatial correlation term based on the Matérn kernel (1|X + Y) to account for the spatial structure of the data considering the centroid of the geographic distribution coordinates (X and Y) of the hosts as spatial variables using the “adespatial” package in R [52]. Additionally, we included a random effect term for host genus (1∣Genus) capturing variation associated with taxonomy, considering that phylogenetically close species are more likely to spill over [53]. The general model specification was as follows:

General model <- fitme (Versatility weighted ~ Zoonotic potential + Body Mass (g) + Feed habit + Matern (1|X + Y) + (1|Genus), family = Gamma (link = “log”).

The models’ fit was assessed based on the Akaike information criterion (AIC) and the relevance of the parameters was determined by the estimated confidence intervals. The mean of the models with ΔAIC ≤ 2 highlighted estimates whose intervals do not include zero, enabling the identification of relevant predictors and discarding those without a significant association with the response variable [54]. In addition, we calculated the Relative Importance Index (RI) of each predictor variable, corresponding to the sum of the Akaike weights in the models in which each predictor was included. Predictors with higher RI indicate greater support within the set of models tested, as they are associated with models with better acceptability.

## 3. Results

### 3.1. Dataset Overview

**Figure 1:**
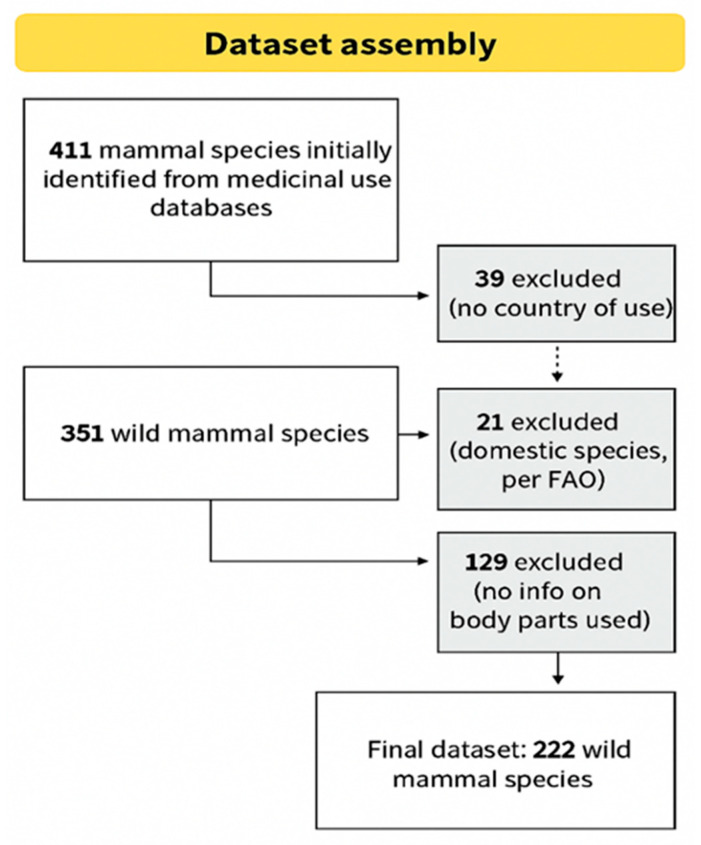
Overview of inclusion and exclusion criteria applied during dataset construction.

From an initial set of 411 mammal species used in traditional medicine, 39 were excluded due to missing information about the country where the use was registered, and 21 were identified as domestic species. With this, we obtained data on the types of zoonotic pathogens (bacteria, fungi, protozoa, viruses, and helminths) associated with 351 species of wild mammals used for medicinal purposes (Table S1). Of these, 129 species (36.7%) lack information on the body parts used in the treatment of diseases, leading to their exclusion from modeling. The final dataset used in the analyses comprised 222 wild mammal species. A flowchart summarizing the data selection process is presented in Figure 1.

### 3.2. Trends in Zoonotic Pathogens Among Medicinal Mammals

**Figure 2:**
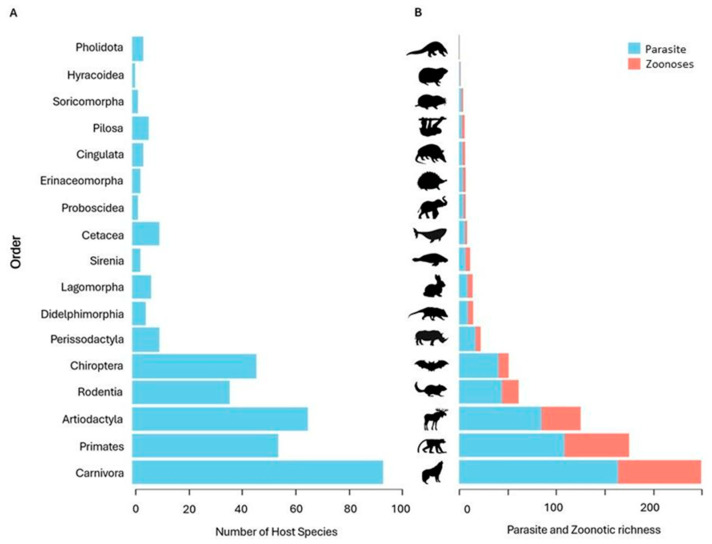
Distribution of host species and richness of zoonotic pathogens among mammalian orders. (**A**) Number of host mammalian species used in traditional medicine, grouped by taxonomic order. (**B**) Total richness of recorded pathogens and number of zoonotic pathogens associated with these species, by mammalian order.

We recorded 411 mammal species used in medicinal practices worldwide, including domestic and wild species. Of these, 351 wild species were analyzed in our study, representing 17 of the 27 existing orders (Figure 2A). The most representative orders were Carnivora (n = 93 species, 26%), Artiodactyla (n = 65, 19%), Primates (n = 54, 15%), Chiroptera (n = 46, 13%) and Rodentia (n = 36, 10%). The other orders comprised the remaining 17%.

**Figure 3:**
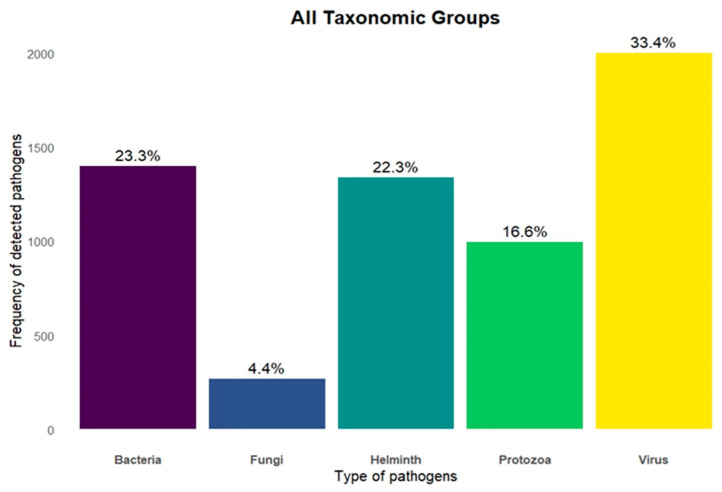
Number of zoonoses associated with the five main groups of pathogens based on their taxonomic classification.

**Figure 4:**
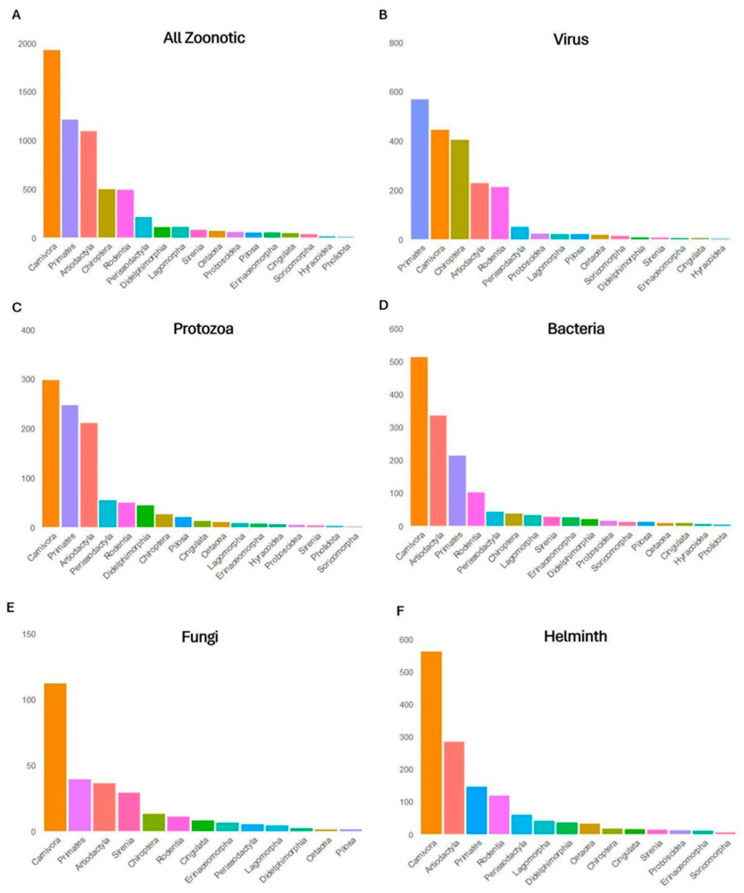
Number of zoonotic diseases attributed to viruses, bacteria, protozoa, helminths and fungi recorded in the 17 taxonomic orders of mammals used in traditional medicine. (**A**) Total number of zoonotic pathogens across all mammal orders (combined pathogen types). (**B**) Number of viruses reported per mammal order. (**C**) Number of protozoa reported per mammal order. (**D**) Number of bacteria reported per mammal order. (**E**) Number of fungi reported per mammal order. (**F**) Number of helminths reported per mammal order.

We identified 5.146 pathogens associated with the 351 mammals analyzed. Of these, 2.778 (53.9%) were classified as zoonotic, as they had already been detected in humans. Among all recorded pathogens, 33% were viruses, 23% bacteria, 22% helminths, 17% protozoa and 4% fungi (Figure 3). The Carnivora order contained the largest proportion of zoonotic pathogens (33.7%), followed by Primates (26.2%), Artiodactyla (15.8%), Rodentia (6.8%) and Chiroptera (4.2%) (Figure 2B). The orders with the lowest zoonotic richness were Pholidota (0.1%), Hyracoidea (0.3%) and Soricomorpha (0.6%) (Figure 2B). The distribution of pathogens varied among taxonomic groups: Primates had the highest number of zoonotic viruses; Carnivora, of helminths; and Artiodactyla, of zoonotic protozoa (Figure 4).

**Figure 5:**
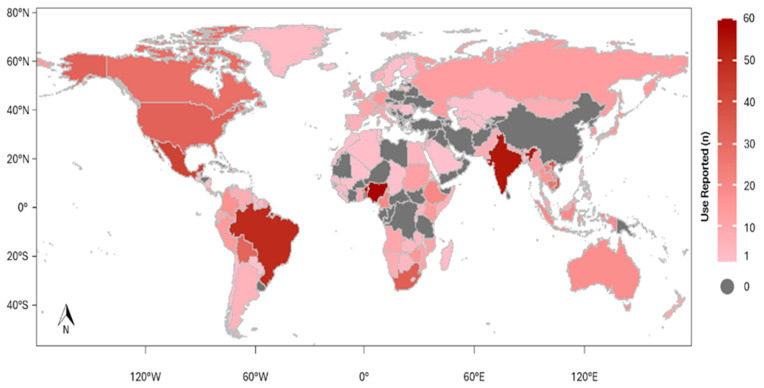
Global occurrence of medicinal use of wild mammals. The map shows countries where at least one medicinal use of a wild mammal species has been reported.

Medicinal use of wild mammals was recorded in 132 of the 195 existing countries. The five countries with the largest number of species used were China (n = 66), India (n = 66), Nigeria (n = 63), Brazil (n = 55) and Benin (n = 53) (Figure 5). The species with the greatest distribution of medicinal use were *Loxondonta africana*—“African Savanna Elephant” (44); *Panthera pardus*—“Leopard” (41); *Ursus arctos*—“Brown Bear” (32); *Ursus thibetanus*—“Asiatic Black Bear” (30); *Melursus ursinus*—“Sloth Bear” and *Panthera tigris*—“Tiger” (29).

The species with the greatest therapeutic versatility based on the RI values (Table 1) were *Ursus arctos*—“Brown bear” (RI = 1.54); *Mephitis macroura*—“Hooded Skunk” (RI = 1.55); *Ursus americanus*—“American Black Bear” (RI = 1.58); *Ursus maritimus*—“Polar Bear” (RI = 1.58); *Dasypus novemcinctus*—“Nine-banded Armadillo” (RI = 1.65); and *Manis gigantea*—“Giant Ground Pangolin” (RI = 1.8).

### 3.3. Determinants of Therapeutic Versatility in Medicinal Mammals

**Figure 6:**
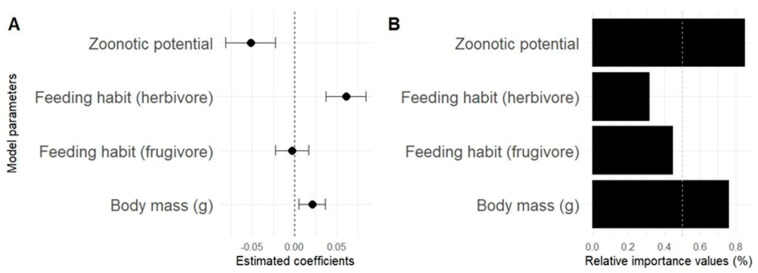
Average of candidate models with ΔAIC ≤ 2 for predicting the therapeutic versatility of mammals. (**A**) Average coefficients (±standard error) of the explanatory variables: zoonotic potential, body mass and feeding habits; (**B**) relative importance of variables (RVI) based on the frequency of inclusion in the models.

When investigating how the therapeutic versatility of mammals relates to zoonotic potential, body mass and feeding habits, we observed that four models presented ΔAIC ≤ 2, indicating equivalent explanatory power. These models include three with a single predictor variable and one with two variables (Table 2), covering all evaluated factors. The results indicate that zoonotic potential presented a negative relationship with therapeutic versatility, with a relative importance (RI) of 60%, being the most influential variable. This suggests that species associated with greater zoonotic risk tend to be less used medicinally (Table 2; Figure 6B). Body mass had a positive relationship with versatility with an RI of approximately 40%, indicating that larger species are more widely used for therapeutic purposes. Although feeding habits were also positive, they had a lesser influence (RI of 20%), suggesting a more limited contribution.

The seven models evaluated presented substantial empirical evidence (Table 2). However, all the explanatory variables included zero overlap within their standard error ranges, indicating limitations in the strength of the associations. For example, the effect of frugivorous eating habits crossed zero (Figure 6A), being considered a non-informative parameter. The null model, with a weight of only 2.9%, had little support, which reinforces the relevance of the variables tested. Zoonotic potential emerged from among the analyzed factors as the main modulator of the versatility of medicinal mammals. The inclusion of this variable in models with greater support, such as the one combining “Zoonotic potential + Body mass (g)”, increased the explanatory capacity, reinforcing its importance as a determining factor in therapeutic versatility.

In terms of predictive hierarchy, zoonotic potential is the main predictor of versatility, followed by body mass and dietary habits, with decreasing influence. Furthermore, while mass and dietary habits showed positive effects, zoonotic potential demonstrated an opposite effect, indicating it as a limiting factor of therapeutic versatility (Table 2).

## 4. Discussion

### 4.1. Trends in Zoonotic Pathogens Among Medicinal Mammals

Our results indicate that mammals used in traditional medicine harbor a wide diversity of zoonotic pathogens. Carnivores, primates and ungulates concentrate the largest number of medicinal species with high zoonotic burden, reflecting both their ecological role and their cultural and medicinal value in different regions of the world [55,56]. These findings reinforce the need to include the therapeutic use of wild mammals in epidemiological surveillance and zoonotic risk management strategies.

Carnivores are often exposed to multiple pathogens due to their generalist diet and high trophic role, acting as reservoirs for helminths, bacteria and fungi [57,58,59,60]. In turn, primates are highly associated with virus transmission, especially due to their phylogenetic proximity to humans, which facilitates bidirectional transmission of pathogens [61,62,63]. Vulnerability to zoonotic transmission by primates is compounded by their widespread use in medicinal contexts. Studies report the presence of simian immunodeficiency virus (SIV) in meat, pets and primate hunters in West Africa [64,65], reinforcing the need for regulation and surveillance. Furthermore, the recent outbreak of Monkeypox (Mpox), even after the COVID-19 pandemic, illustrates the persistent threat of zoonoses emerging through human interaction with and exploitation of wildlife [66].

Large mammals also stand out as species with high therapeutic versatility and a high zoonotic load. For example, bears are hosts to several infectious agents, such as Alphacoronavirus 1, which can infect humans [67]. The nine-banded armadillo (*Dasypus novemcinctus*) is associated with the transmission of *Mycobacterium leprae*, which causes leprosy [68,69]. The pangolin (*Manis gigantea*) is widely used in different Asian cultures, and has been associated with the wildlife trade and concerns regarding its potential role in the transmission of emerging infectious diseases, although specific data on this species are lacking [70,71,72].

Viruses and bacteria were the most prevalent among the types of pathogens among mammals used in medicine, constituting a pattern consistent with the recent history of health emergencies such as HIV, SARS, MERS, Ebola, H1N1, and COVID-19 [73,74,75]. In this context, traditional medicine should be considered a relevant exposure route to zoonoses, especially in widely used taxonomic groups, such as primates and carnivores. Because the latter occupy high trophic levels and consume several species, they act as important “pathogen deposits” [56,76] and present a high diversity of bacteria, fungi and helminths in our data. On the other hand, primates mainly stood out as viral reservoirs, reflecting already documented patterns in the literature [77,78].

Despite these risks, little attention has been given to the management of wild animals used in traditional medicine and the potential associated health impacts [4,11,79,80]. Large species, such as ungulates, not only deserve attention because of the zoonotic risk, but also because they are widely exploited due to the abundance of biomass and proximity to humans and domesticated animals, which can favor pathogen sharing [35,81]. In addition to the considerable volume of tissue and organs available, their larger body size is generally associated with greater mobility and geographic distribution, constituting factors which increase the interface with human populations, and therefore increase the risk of zoonosis transmission, especially when compared to smaller species, such as rodents.

The global usage distribution of medicinal mammals with records in 132 countries reflects a strong cultural dependence on these resources, especially in regions with rich biodiversity and consolidated traditions of traditional medicine, such as Asia, Latin America and Africa. More specifically, China, India, Nigeria, Brazil and Benin lead in the number of species used, indicating the relationship between biological diversity and cultural history. These findings reinforce the importance of local and regional analyses to identify usage patterns and guide conservation strategies adapted to sociocultural contexts.

### 4.2. Determinants of Therapeutic Versatility in Medicinal Mammals

Our results show that zoonotic potential exerts a negative influence on the therapeutic versatility of medicinal mammals, indicating that empirical perceptions of health risk shape species selection practices. Although anchored in deep cultural traditions, these practices appear to integrate adaptive strategies aimed at minimizing risks, even in the absence of formal biomedical knowledge [82]. This pattern can be interpreted in light of evolutionary theories of pathogen avoidance behavior, such as the “behavioral immune system,” in which emotional responses such as disgust, aversion, and fear function as psychological defenses against exposure to infectious agents [30,31,83]. Thus, considering that many traditional medical systems have developed over millennia with direct interaction with wildlife, it is plausible to assume that zoonotic risk has directly or indirectly influenced the selection of the most appropriate species for therapeutic use. Wild animals take on functions in traditional medicine which go beyond the simple treatment of illnesses, incorporating symbolic, social and spiritual values. Products such as pangolin meat, rhino horns and shark fins continue to be valued, even with a high zoonotic risk [6,84]. This persistence shows that the choice of medicinal resources operates in a multifactorial system, where therapeutic efficacy, social status and symbolic value often override perceptions of risk.

The use of wild animals for medicinal purposes, often as a byproduct of hunting [85,86,87], involves different zoonotic risks, depending on the species and the product handled. Tissues such as the liver, kidneys and brain concentrate a high pathogenic load, including viruses such as Ebola in primates [88], bacteria such as *Brucella abortus* in ungulates [89,90] and coronaviruses in bats [91]. These examples illustrate how decisions about which species and parts to use may not only reflect cultural preferences, but also adaptive strategies (albeit implicit) to minimize health risks.

From an evolutionary perspective, it would be disadvantageous to select species whose use entailed high risks, reducing the potential benefits of the resource [92,93]. Thus, it would be expected that animals with greater zoonotic potential would be avoided. However, traditional medical systems operate under multiple factors, which are not always linear or utilitarian, and are influenced by different biocultural and ecological factors [94]. Studies on selection patterns demonstrate that organoleptic properties, such as taste and odor, influence selection of medicinal species [95,96,97]. In addition, perceptions of risk and toxicity are also considered, which may lead to excluding or restricting certain species [98,99].

In addition to zoonotic potential, factors such as body mass and feeding habits also influence therapeutic versatility, although to a lesser extent. Larger species tend to be used more, which may reflect both their greater yield in terms of biomass (organs, fat, bones) and a symbolic perception of strength or therapeutic efficacy [82,100,101]. However, this preference can be contradictory from a health point of view, as larger animals often host a greater diversity of pathogens, increasing the risk of zoonotic transmission [102,103]. Herbivorous and frugivorous species showed a slight tendency to be more versatile regarding eating habits, possibly due to cultural value and positive symbolic associations with purity, balance and health [104,105,106].

The integrated analysis of statistical models highlights that versatility results from the interaction between ecological, cultural and health risk factors. Zoonotic potential stands out as a significant barrier to use, so that species associated with a higher risk of transmission tend to be less versatile from a medicinal point of view and thus less used, reflecting an empirical logic of risk aversion. This trend suggests an interface between traditional knowledge and perceptions of safety that, even if not formalized, can act as filters in the selection process [107]. Communities have developed cultural strategies, such as cooking and smoking animals, to reduce biological risks, although these methods do not completely eliminate pathogens [108,109]. This reinforces the need to integrate traditional and scientific knowledge to promote safer practices that are compatible with biodiversity conservation.

### 4.3. Health and Conservation Implications and Study Limitations

The results of this study reinforce that the medicinal use of wild mammals represents a complex interface between public health, biodiversity conservation and sociocultural systems. The high diversity of species involved and the selection patterns observed indicate that although traditional medicinal practices are culturally legitimate, they can generate significant impacts on animal populations and on ecological balance, especially when associated with unregulated exploitation.

Overexploitation for medicinal purposes can contribute to the population decline of already vulnerable species, intensifying the risk of local and regional extinction [110,111]. Furthermore, as many of these mammals perform fundamental ecological functions, such as seed dispersal [112,113,114], the control of invertebrate populations [114] and the maintenance of the trophic structure [115]; their reduction can directly affect the integrity of ecosystems. Therefore, understanding the usage patterns and the factors which influence selection of these species is an essential step to support public policies for sustainable management and health surveillance [34,116].

Our results also highlight the need to incorporate the zoonotic dimension into conservation strategies. When species with a high potential for pathogen transmission are used in traditional contexts without adequate hygiene or processing measures, they represent a risk pathway for the health of human communities, especially those that directly depend on wildlife for subsistence [13,117,118]. Integrating ecological, ethnozoological and health data can serve as a basis for more integrated action plans aimed at both biodiversity protection and the safety of human populations. Formulated surveillance policies should consider the ecological and geographic context in which species occur. In addition, measures such as educational campaigns, review of management practices or the proposal of safe therapeutic alternatives can be essential to mitigate risks in areas where certain species overlap with vulnerable human communities. Localized strategies based on scientific evidence and intercultural dialogue are especially relevant given the complexity of traditional medical systems.

The disparity in the distribution and accessibility of species also influences their usage pressure. Species widely available in certain regions, such as *Loxodonta africana* and *Panthera pardus*, are associated with multiple medicinal uses with the use of various body parts (e.g., fat, stomach, skin, teeth, testicles and kidneys), which can intensify their extraction pressure [119,120,121]. In contrast, species with more restricted distribution or low population density, such as *Spilogale gracilis* and *Paradoxurus hermaphroditus*, tend to be less used, possibly due to their lower local availability [122,123,124]. The scarcity of ethnobiological records related to these species reinforces the hypothesis that ecological accessibility—determined by abundance and distribution—directly influences their inclusion or exclusion in medicinal practices. This disparity in use combined with the scarcity of data in some regions can mask critical exploitation patterns and compromise the effectiveness of conservation actions.

As with all studies based on secondary data, this analysis has inherent limitations in the quality and scope of the information available. The taxonomic, geographic and ecological accuracy of records may vary between sources and countries, reflecting both inequalities in monitoring capacity and knowledge gaps in less-studied regions [125]. Furthermore, by assuming that pathogens associated with a host species are uniformly distributed throughout its range (as defined by the IUCN), we disregard contextual factors important to transmission dynamics, such as the presence of vectors, local environmental conditions, seasonality and population density [126,127]. Although this simplification is methodologically necessary to enable analyses on a global scale, it represents an important limitation to be considered, especially when interpreting the results in regional and local contexts.

## 5. Conclusions

This study demonstrated that the therapeutic versatility of mammals used in traditional medicine is influenced by a combination of ecological, health and cultural factors. Zoonotic potential emerged as the main limiting factor, indicating that species with a higher risk of disease transmission tend to be avoided, even when they have multiple therapeutic applications. Factors such as body mass and dietary habits also contributed to explaining versatility, reflecting practical criteria for use and symbolic values associated with the species. These findings highlight the importance of local and regional analyses which reveal specific usage patterns, help identify species at greater risk and guide culturally sensitive conservation strategies. Thus, it becomes possible to develop more effective interventions by integrating ecological, health and sociocultural aspects to both preserve biodiversity and to protect the health of human communities that depend on it.

## Figures and Tables

**Table 1:** Medicinal mammal species with the greatest therapeutic versatility, ordered by relative importance (RI).

Species	Order	RI	Body Part Used	Virus (n)	Protozoa (n)	Bacteria (n)	Fungi (n)	Helminths (n)
*Ursus arctos*	Carnivora	1.54	Unspecified	7	2	5	2	12
*Mephitis * *macroura*	Carnivora	1.55	Meat, fat	1	0	0	0	0
*Ursus* *americanus*	Carnivora	1.58	Unspecified	9	2	3	1	12
*Ursus maritimus*	Carnivora	1.58	Blood, Carapace, Nails, Fat, Lard, Feet, Flesh, Skin, Tail, Urine, Whole Animal	6	4	1	1	4
*Dasypus* *novemcinctus*	Cingulata	1.66	Blood, Carapace, Nails, Fat, Lard, Feet, Meat, Skin, Tail, Urine, Whole Animal	3	3	1	0	2
*Manis gigantea*	Pholidota	1.8	Blood, Bones, Brain, Eyes, Fat, Flesh, Head, Scales, Whole animal, Body, Claws, Meat, Scales	0	0	1	0	0

Mammalian species with IR > 5. Therapeutic versatility is expressed through the Relative Importance Index (RI), which considers both the number of use categories (NUC) and the number of therapeutic indications (NT) reported for each species.

**Table 2:** Candidate models with ΔAIC ≤ 2 explaining the therapeutic versatility of medicinal mammals based on zoonotic potential, body mass and feeding habits.

Model	k	LogLik	AICc	Δ	Weight
1. Zoonotic potential	0.295	367.191	−720.381	0.000	0.295
2. Body mass (g)	0.169	366.635	−719.271	1.110	0.169
3. Feeding habitat	0.152	367.529	−719.058	1.323	0.152
4. Zoonotic potential + body mass (g)	0.146	369.487	−718.974	1.407	0.146
5. Zoonotic potential + feeding habit	0.090	370.003	−718.006	2.375	0.090
6. Body mass (g) + feeding habitat	0.068	369.732	−717.464	2.917	0.068
Gamma (zoonotic potential + body mass (g) + feeding habit)	0.048	370.380	−716.761	3.620	0.048
Null	0.029	362.891	−715.783	4.598	0.029

Models with substantial empirical evidence (ΔAIC ≤ 2); k = number of parameters in the model; Loglik = log-likelihood; AIC = second-order Akaike information criterion; Δ = AIC difference between the model under concern and the best model; Weight = Akaike weight, i.e., the likelihood of the model being the best in the set of candidate models.

## Data Availability

The original contributions presented in this study are included in the article. Further inquiries can be directed to the corresponding author.

## Supplementary Materials

The following supporting information can be downloaded at https://www.mdpi.com/article/10.3390/pathogens14070640/s1. Table S1. Wild mammals with medicinal uses recorded globally.
